# The association between e-cigarette use and sleep health: A systematic review and meta-analysis

**DOI:** 10.18332/tid/225458

**Published:** 2026-09-18

**Authors:** Ishnaa Gulati, Sashini Kosgoda, Anasua Kundu, Robert Schwartz, Apsara Ali Nathwani, Jennifer Soer, Jessie Tian, Michael Chaiton

**Affiliations:** 1 Dalla Lana School of Public Health, University of Toronto, Toronto, Canada; 2 Centre for Addiction and Mental Health, Toronto, Canada; 3 Institute of Health Policy, Management and Evaluation, University of Toronto, Toronto, Canada; 4 Institute of Medical Science, University of Toronto, Toronto, Canada

**Keywords:** e-cigarette, electronic cigarette, sleep, meta-analysis, systematic review

## Abstract

**INTRODUCTION:**

E-cigarette use has raised concerns about health impacts beyond nicotine dependence, yet evidence on the relationship between vaping and sleep disturbance remains limited. This systematic review and meta-analysis examined the association between exclusive e-cigarette use and sleep health.

**METHODS:**

MEDLINE, Embase and PsycINFO were searched up to July 2025 for English-language, peer reviewed human studies. Eligible studies were peer-reviewed observational studies of people who used nicotine e-cigarettes without combustible cigarette use, compared with non-users, that reported sleep health outcomes such as sleep duration and quality. Two independent reviewers screened all records, extracted data using the STROBE checklist, and assessed quality with the Joanna Briggs Institute Critical Appraisal tool. Effect sizes were pooled using random-effects models, and odds ratios (ORs) and 95% confidence intervals (CIs) are reported.

**RESULTS:**

Sixteen cross-sectional studies met inclusion criteria, of which twelve were included in the meta-analysis, most of moderate quality. Past 30-day exclusive e-cigarette users had a 38% greater likelihood of poor sleep health than never users (OR=1.38; 95% CI: 1.24–1.55), with similar associations in adolescents (OR=1.32; 95% CI: 1.06–1.66) and adults (OR=1.36; 95% CI: 1.15–1.61).

**CONCLUSIONS:**

E-cigarette use was associated with poorer sleep health, with similar associations in adolescents and adults. Given rising youth vaping, longitudinal studies with objective measures are needed to inform public health strategies.

## INTRODUCTION

Sleep is a fundamental biological process essential for cognitive function, emotional regulation, and overall physical well-being^1^. It involves multiple stages, each contributing uniquely to the body’s restoration and functionality^1^. Disruptions to normal sleep architecture can have far-reaching consequences, extending beyond immediate fatigue to include impaired attention, concentration, and decision-making abilities, as well as an increased risk of chronic health conditions, such as cardiovascular diseases, metabolic disorders, and mental health disorders^2^.

Nicotine exposure is a well-established risk factor for sleep disturbances^3^. Prior research on combustible cigarette use has consistently shown associations between nicotine intake and prolonged sleep latency and poorer sleep quality. Higher doses of nicotine have been also found to be associated with decreased REM sleep and less hours of sleep time^4^. E-cigarettes, also known as Electronic Nicotine Delivery Systems (ENDS), have emerged as a popular alternative to traditional cigarettes since their introduction in 2004^5^. These battery-operated devices heat a liquid solution containing nicotine and various other additives^5^. Although initially marketed as safer smoking-cessation tools, manufacturers have employed novel marketing approaches and terminology^6^ such as ‘vaping device’ or ‘hookah pen’ to distance their products from the negative connotations of traditional cigarettes. Many e-cigarette companies promote these products as cleaner, healthier and more affordable alternatives to traditional smoking^7,8^. Such perceptions, combined with aggressive marketing and the introduction of appealing flavors, have encouraged widespread adoption of e-cigarettes even among never smokers, particularly youth and young adults^9,10^.

This rapid rise in e-cigarette use has raised new questions about its potential impact on sleep health. Nicotine, the primary psychoactive component of e-cigarettes, stimulates cholinergic neurotransmission by activating α7 and α4β2 nicotinic Acetylcholine (ACh) receptors, indirectly affecting glutamatergic, dopaminergic, and serotonergic pathways involved in arousal and sleep regulation^3^. Beyond that, the harmful chemicals present in e-cigarette aerosols, including flavoring agents and other additives, may add additional layers of complexity to this association and further disrupt sleep physiology^11^.

A few reviews have also identified sleep as a potential health outcome related to e-cigarette use but consistently highlight sparse and inconsistent evidence^12,13^. Some studies report that e-cigarette users experience poorer sleep quality or shorter sleep duration^14,15^, while others find no significant association^16^. To address these gaps, the present systematic review and meta-analysis synthesize existing literature to measure the independent impact of e-cigarette use on sleep health.

## METHODS

### Data sources and searches

This systematic review was conducted by following the methodology of a previous project ‘Vaping and Electronic Cigarette Toxicity Overview and Recommendations (VECTOR)’, where we conducted separate reviews to assess major health effects (i.e. cardiovascular, respiratory, cancer, dependence) of e-cigarette use^17-19^. The protocol of VECTOR reviews was prospectively registered in PROSPERO (CRD42023385632). While we followed the same methodology, we made some content replacements such as focusing on the association between e-cigarette use and sleep health rather than the four major health outcomes assessed in the VECTOR project, and excluding former smokers and dual users as comparison groups. We adhered to the Preferred Reporting Items for Systematic Reviews and Meta-Analyses (PRISMA) guidelines for a transparent reporting process. Our comprehensive search strategy included EMBASE, MEDLINE, and PsycINFO databases via Ovid from the inception of the databases up to 31 July 2025, for a thorough exploration of the existing literature. An initial search of the literature was conducted on 16 October 2023, and the search was later updated on 31 July 2025. The search terms were derived from a predefined set of keywords, as detailed in the Supplementary file Information 1, providing a standardized and systematic approach to identify relevant studies for inclusion in this review. By following the PRISMA guidelines and a careful search strategy, this systematic review aimed to contribute a robust and unbiased synthesis of the current evidence on the relationship between e-cigarette use and sleep health.

### Study selection

Search results were uploaded into Covidence, a systematic review software package, where duplicate articles were automatically removed. Two independent reviewers screened the titles, abstracts, and full texts for eligibility among all identified articles (up to July 2025). Any disagreements were resolved through discussions between the reviewers. Only studies published in English and involving human participants were eligible for inclusion. Grey literature was excluded from the review.

To ensure a focused examination of e-cigarette-related sleep health outcomes, only studies including individuals who used nicotine e-cigarettes, but not combustible cigarettes, were included. E-cigarette use included the use of only nicotine vape pens, e-hookahs, cigalikes, mods, JUUL, tank systems, electronic vapor products, electronic nicotine delivery systems, and/or alternative nicotine delivery systems.

Other inclusion criteria for this systematic review encompassed peer-reviewed research articles with robust methodologies that investigated the relationship between e-cigarette use and sleep health. Specifically, eligible studies must provide information on various aspects of sleep health, including duration and quality and to clearly specify e-cigarette use as nicotine-containing electronic devices. Conversely, exclusion criteria included abstracts, conference proceedings, editorials, author responses, theses, and books, as these may lack the depth of data required for comprehensive analysis. Studies were also excluded if they have insufficient outcome data, misidentification of e-cigarette exposure such as failing to differentiate between nicotine, no nicotine and cannabis vaping, ineligible outcomes, and those lacking specification about participants’ smoking status were also excluded.

### Data extraction

Two independent reviewers extracted data from included studies using the STROBE checklist^20^, with disagreements resolved by consensus. Extracted data included study characteristics (title, background, objective, location of which the study was conducted, study design, setting, duration of the study), participant characteristics (population description, inclusion and exclusion criteria, method of recruitment and bias involved, total participants), outcome, exposure and comparison group, statistical methods and potential confounders, key findings and statistical significance of the reported findings. Exposure was measured as self-reported use of nicotine e-cigarette in the past 30-days, every day or somedays, or past year. Comparison groups included non-e-cigarette users, never e-cigarette users, and exclusive cigarette smokers. Extracted outcomes of primary interest were any type of sleep disturbance, including sleep quality and sleep duration. Limitations, interpretations, generalizability, and funding were also extracted^20^. A summary of the extracted data can be found in the Supplementary file Information 2.

### Quality assessment

The Joanna Briggs Institute (JBI) critical appraisal checklist was used as the preferred tool for quality assessment^21^. The tool was selected because of its recognized suitability for evaluating observational study designs. Two independent reviewers conducted the quality assessment separately and any disagreement between the reviewers were resolved by consensus. Each of 8 items was rated as 1 if the response to the item was ‘yes’, and 0 if it was ‘no’ or ‘unclear’. We followed the Mansoor and Khuwaja^22^ study and categorized the studies into low, moderate and high quality if the final rating on JBI critical appraisal scale was 1–3, 4–6, and 7–8, respectively.

### Statistical analysis

We considered a study eligible for meta-analysis if the study assessed the impact of e-cigarette use on overall sleep health among past 30-day exclusive e-cigarette users and never users. Instead of assessing individual associations on different sleep related outcomes (i.e. sleep duration, sleep quality), we conducted a single meta-analysis to assess the risk of poor sleep health which encompasses any type of sleep disturbance. This approach was adopted to ensure that an adequate number of studies were available for quantitative synthesis. Past 30-day e-cigarette use included self-reported use of e-cigarette in the past 30-days, or past month, or every day or somedays. While never users were defined as never using e-cigarettes in their lifetime. Studies were excluded from meta-analysis if they reported non-comparable effect sizes. We could not conduct any assessment of the impact on sleep health between past 30-day e-cigarette users and exclusive cigarette smokers due to the low number of studies meeting the criteria and heterogeneity between the studies. For studies that reported multiple adjusted models, the effect sizes adjusted for potential confounders, including demographic, socioeconomic, and health-related variables, were selected to make them comparable. All studies included in the meta-analysis reported effect sizes as odds ratios (ORs), with the exception of one study^23^, which reported a prevalence ratios (PRs). Log-transformed effect sizes were pooled using a random-effects model, and the overall effect estimate was reported as an OR. Forest plots were created to summarize information from individual studies. We considered statistically significant results in the meta-analysis when p<0.05 or 95% CI did not cross the null value. Heterogeneity was assessed primarily using the I2 statistics, with values of <25%, 25–50%, and >50% considered to indicate low, moderate, and high heterogeneity, respectively^24^. Separate subgroup analyses were performed for adults (aged >18 years) and adolescents (aged ≤18 years) to investigate potential differences in the associations of e-cigarette use on sleep health between the two populations. In addition, a meta-regression was conducted to formally test if age group moderated the association between e-cigarette use and sleep outcomes.

Furthermore, to assess the robustness of the findings, a leave-one-out sensitivity analysis was conducted by excluding each study iteratively to evaluate the impact on the overall effect size^25^. This approach also assessed whether removing the study^23^ reporting PR as effect size affected the pooled estimate. Funnel plots were examined to assess potential publication bias. Asymmetry was further assessed using Egger’s test^26^. All analyses were performed using RStudio Version 2025.06.0.

## RESULTS

### Overview of the included studies

**Figure 1:**
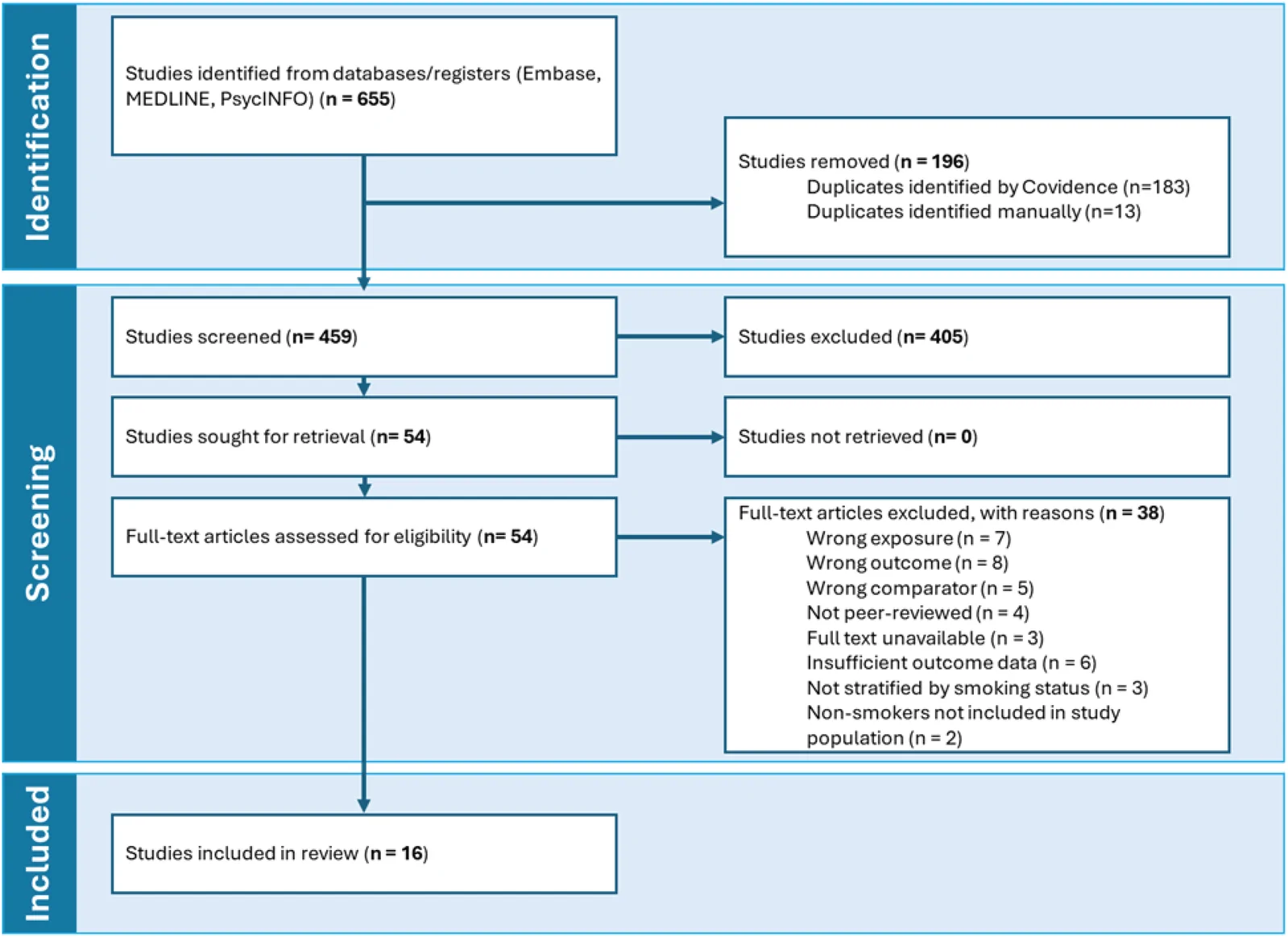
PRISMA flow diagram of included cross-sectional studies assessing the association of e-cigarettes on sleep health, databases search up to July 2025 (N=16)

We identified 655 studies in our search that were potentially eligible. After removing 196 duplicates, 459 titles and abstracts remained. Among these, 54 articles were screened via full-texts, and ultimately, 16 studies were included in this systematic review and meta-analysis (Figure 1).

All 16 included studies^14,15,23,27-39^ were cross-sectional, with most (13) conducted in the United States. The study by Advani et al.^33^ utilized pooled data from 47 countries. Data collection across the included studies spanned from 2013 to 2024, involving a total of 150730 participants (Supplementary file Information 2).

Of the 16 studies, 8 studies^14,15,23,27,34,35,38,39^ were conducted in adults (aged >18 years), 5 studies^28-31,36^ in adolescents (aged ≤18 years), and 3 studies^32,33,37^ in both adolescent and adult population.

The included studies assessed a range of sleep-related outcomes, including sleep disturbances, poor sleep quality, inadequate duration and failure to meet the recommended 7–9 hours of good quality sleep per night^40^. All 16 studies adjusted for potential confounders, including demographic, socioeconomic, and health-related variables, in their final analyses. Quality assessment scores of the studies ranged from 3 to 8, with 13 studies rated as moderate quality, two as high quality, and one as low quality (Table 1).

**Table 1:** Quality scores of the cross-sectional studies based on Joanna Briggs Institute (JBI) critical appraisal tool for analytical cross-sectional studies (N=16)

*Authors (Year)*	*1*	*2*	*3*	*4*	*5*	*6*	*7*	*8*	*Score*	*Quality*
Wiener et al.^27^ (2020)	U	N	N	N	Y	Y	Y	Y	4	Moderate
Merianos et al.^14^ (2023)	U	Y	U	U	Y	Y	Y	Y	5	Moderate
Dunbar et al.^28^ (2017)	U	N	N	N	Y	Y	N	Y	3	Low
Riehm et al.^29^ (2019)	U	Y	N	N	Y	Y	N	Y	4	Moderate
Brett et al.^15^ (2020)	Y	Y	N	Y	Y	Y	Y	Y	7	High
Jackson et al.^30^ (2020)	U	Y	N	N	Y	Y	Y	Y	5	Moderate
Kianersi et al.^23^ (2021)	Y	Y	U	U	Y	Y	Y	Y	6	Moderate
Merianos et al.^31^ (2021)	U	Y	N	N	Y	Y	Y	Y	5	Moderate
Benyo et al.^32^ (2021)	Y	Y	Y	N	Y	Y	N	N	5	Moderate
Advani et al.^33^ (2022)	Y	Y	Y	Y	Y	Y	Y	Y	8	High
Smucker et al.^34^ (2023)	Y	Y	U	U	Y	Y	N	Y	5	Moderate
Baiden et al.^36^ (2023)	Y	Y	Y	N	Y	Y	N	Y	6	Moderate
Christian et al.^35^ (2023)	U	U	Y	U	Y	Y	Y	Y	5	Moderate
Li et al.^37^ (2024)	Y	Y	U	N	Y	Y	N	Y	5	Moderate
Poudel et al.^39^ (2024)	Y	U	Y	N	Y	Y	N	Y	5	Moderate
Wang et al.^38^ (2024)	U	Y	N	U	Y	Y	Y	N	5	Moderate

Rating score is from 1 (lowest) to 8 (highest). Y: yes. N: no. U: unclear. Meeting Criteria: 1) Were the criteria for inclusion in the sample clearly defined?; 2) Were the study subjects and the setting described in detail?; 3) Was the exposure measured in a valid and reliable way?; 4) Were objective, standard criteria used for measurement of the condition?; 5) Were confounding factors identified?; 6) Were strategies to deal with confounding factors stated?; 7) Were the outcomes measured in a valid and reliable way?; and 8) Was appropriate statistical analysis used? Quality scores were categorized^22^ into groups: Low 1–3, Moderate 4–6, and High 7–8.

### Meta-analysis findings

**Figure 2:**
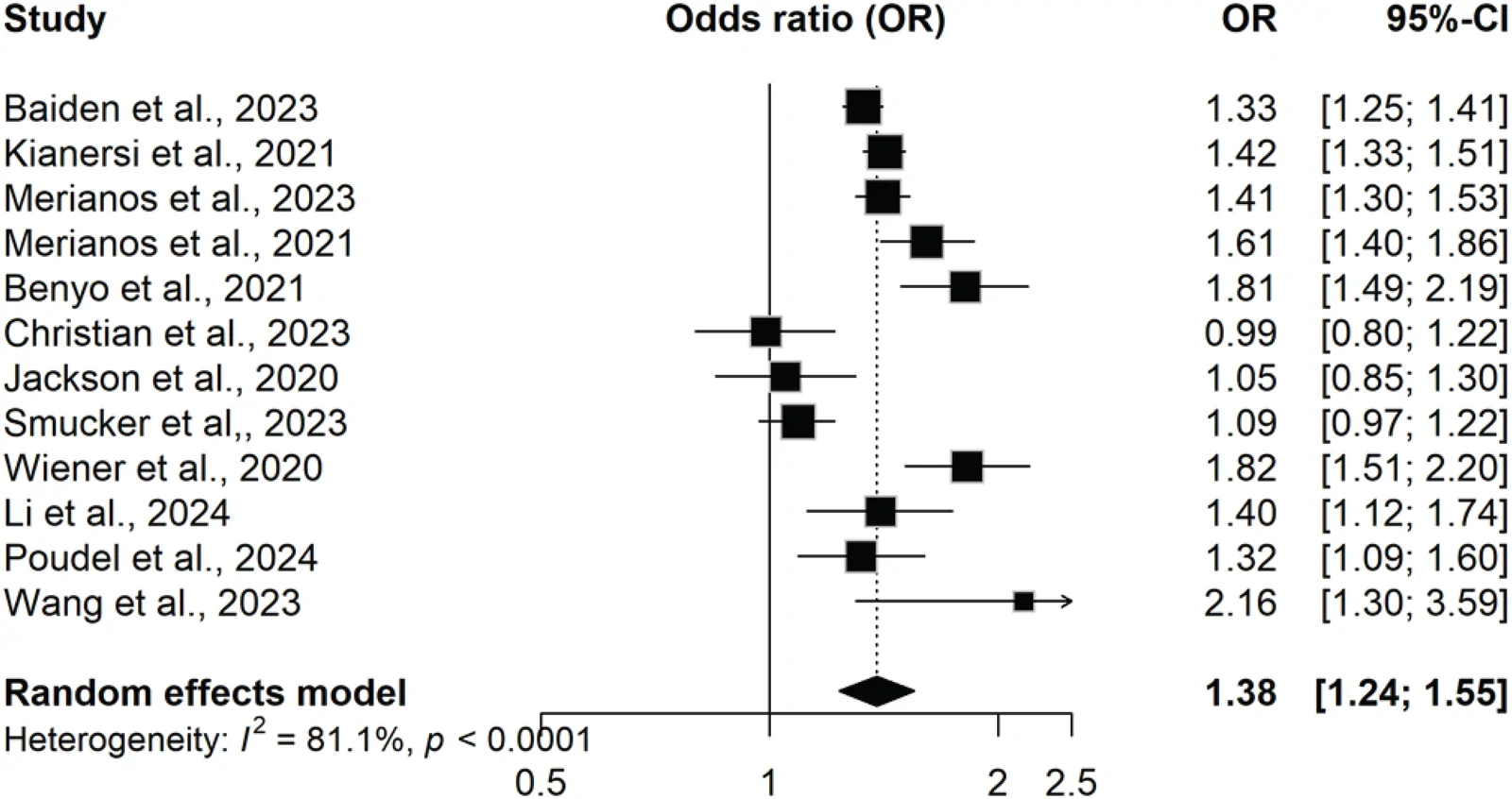
Forest plot displaying pooled odds ratios (ORs) with 95% confidence intervals (CIs) for poor sleep health among past 30-day exclusive e-cigarette users compared to never users, databases search up to July 2025 (N=12). Estimates were pooled using random effects model

Of the 16 studies, 12 studies met the eligibility criteria for inclusion in the meta-analysis. The pooled analysis of 12 studies which compared risk of poor sleep health between past 30-day e-cigarette users and never users showed an overall statistically significant association with a pooled odds ratio (OR) of 1.38 (95% CI: 1.24–1.55) (Figure 2). However, significant heterogeneity was observed among the studies (p<0.0001; I^2^=81.1%) in the meta-analysis. The funnel plot appeared symmetrical (Supplementary file Figure S1), and Egger’s test did not detect significant publication bias (t=0.28, df=10, p=0.783).

*Subgroup analysis by age*

**Figure 3:**
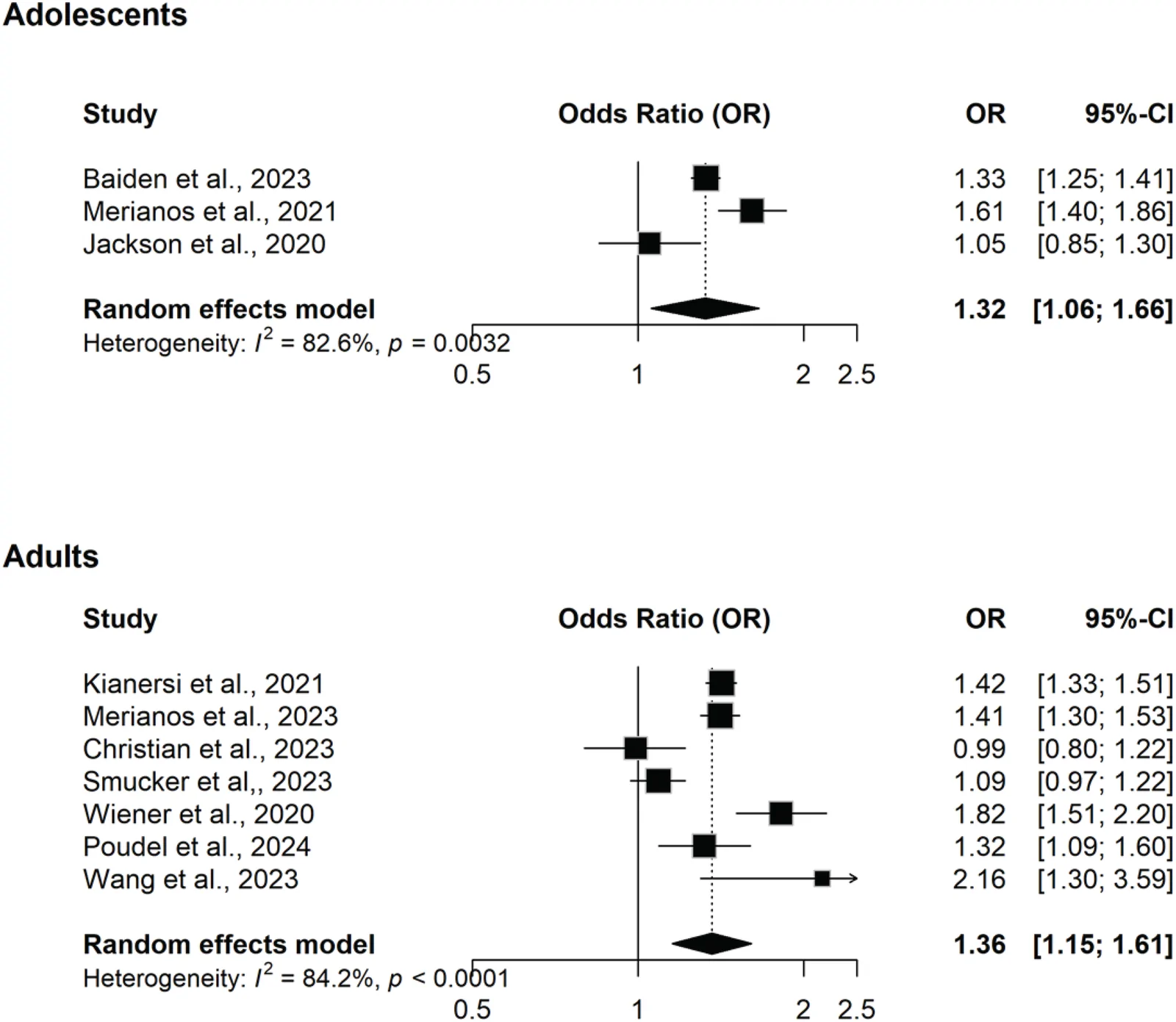
Forest plot of pooled odds ratios (ORs) with 95% confidence intervals (CIs) for poor sleep health among adolescents (N=3) and adults (N=7) past 30-day e-cigarette users versus never users. Estimates with pooled using random effects model

Of the 12 studies included in the meta-analysis, three studies focused on the adolescent population^30,31,36^ and seven studies were conducted in adults^14,23,27,34,35,38,39^. Two studies^32,37^ included both adolescents and adults as study participants and were therefore excluded from the subgroup analysis. For both populations, the pooled analyses showed a statistically significant association between e-cigarette use and poor sleep health with an OR of 1.32 (95% CI: 1.06–1.66) among adolescents and OR of 1.36 (95% CI: 1.15–1.61) among adults (Figure 3). Both subgroup analyses showed substantial heterogeneity (p<0.0032; I^2^=82.6% for adolescents and p<0.0001; I^2^=84.2% for adults). A meta-regression analysis testing age group as a moderator was not statistically significant (Q-test for moderators: QM [df=1] = 0.04, p=0.844) (Supplementary file Information 3).

*Sensitivity analysis*

A sensitivity analysis, excluding each study one at a time, demonstrated the influence of individual studies on the overall effect estimate. The pooled effect sizes remained stable, ranging from OR from 1.35 to 1.42, with 95% CI ranging between 1.21 and 1.59 (Supplementary file Figure S2 and Information 4). While no individual study significantly contributed to heterogeneity, it is noteworthy that excluding Benyo et al.^32^ and Weiner et al.^27^ resulted in the smallest pooled OR, whereas exclusion of Christian et al.^35^, Smucker et al.^34^, and Jackson et al.^30^ yielded the highest pooled estimate.

### Other findings

Four studies^15,28,29,33^ were excluded from the meta-analysis due to not meeting eligibility criteria of meta-analysis. Three studies reported associations consistent with our meta-analysis findings, linking e-cigarette use to poorer sleep outcomes, including shorter sleep duration, lower sleep quality, and greater sleep-related problems and complaints. One study did not find any statistically significant association between e-cigarette use and sleep latency or quality^33^.

## DISCUSSION

The current systematic review and meta-analysis summarized and quantified the association between e-cigarette use and sleep health, indicating that users had 38% higher odds of experiencing sleep-related problems compared to never users. To our knowledge, this is among the first comprehensive quantitative syntheses to examine this. Distinct from the prior investigations, the present analysis focused exclusively on e-cigarette users, excluding dual and cigarette smokers, thereby providing a clear interpretation of the independent associations of vaping on sleep health. By using data from 16 cross-sectional studies involving over 150000 participants, our findings provide robust evidence supporting an adverse impact of e-cigarette use on overall sleep health.

Building upon prior research that has established association between substances such as alcohol, marijuana, and cigarettes and disturbances in sleep patterns, this study adds to the existing body of knowledge by identifying a comparable relationship between the use of e-cigarettes and sleep health. The stimulant nature of nicotine, a key component in e-cigarettes, is likely a major contributor to this association^41^. Nicotine’s impact on the central nervous system can lead to difficulties in both initiating and maintaining sleep compared to those who do not vape^41^.

We found approximately greater risk of poor sleep health among adolescent and adult past 30-day e-cigarettes users, respectively. Moreover, our meta-regression analysis suggested that the difference in effect size between adolescents and adults was not statistically significant, emphasizing the consistent association across both groups. This finding aligns with two existing meta-analyses that have explored sleep and substance use relationships, reinforcing that e-cigarette use poses comparable risks to sleep health across age groups^42,43^. Our findings further highlight the distinct vulnerability of adolescents to the adverse effects of nicotine exposure^3,44,45^. The developing neurobiological environment of youth may heighten sensitivity to nicotine’s sleep-disturbing properties compared to adults^46^, which could explain the observed impact of poor sleep health among adolescent e-cigarette users. Psychosocial stressors such as academic pressure, societal expectations, and peer influences may further worsen this vulnerability^47^. This is consistent with previous evidence linking e-cigarette use to mental health challenges and poorer sleep quality among youth^13^. However, our findings on adolescents are based on only 3 studies. Hence, further research is needed to examine this association between e-cigarette use and adolescent sleep health.

Similarly, among adults the link between e-cigarette use with poor sleep has important implications, especially since e-cigarettes are often used as smoking cessation aids^48^. Nicotine, regardless of delivery method, disrupts sleep and can impair cognition, elevate, stress and cravings and reduce the likelihood of successful quitting^49^. Although, we could not conduct a meta-analysis comparing exclusive e-cigarette users and cigarette users, several studies in our review found no significant difference in sleep outcomes between the two groups, suggesting that e-cigarette use may not offer meaningful sleep-related advantages over smoking^14,27-29^.

In light of these findings, it is important to address the public health implications associated with the observed association between e-cigarette use and compromised sleep health. The documented impact on sleep among individuals who vape e-cigarettes raises concerns about the broader well-being of communities. As sleep plays a vital role in physical and mental health, the potential consequences of widespread e-cigarette use on public health cannot be overstated, especially among youth and young adults^10,50^. Addressing this issue requires a multifaceted approach, including targeted public health campaigns, educational initiatives, and regulatory actions to discourage youth e-cigarette use, including restrictions on advertising campaigns and limitations on characterizing flavors^51^. Furthermore, healthcare professionals should be equipped with the knowledge to counsel patients on the potential risks of e-cigarette use and maintaining healthy sleep patterns^52^. Future studies, especially longitudinal studies considering potential confounders, such as mental health, stress, or behavioral factors, and incorporating objective measures of sleep are crucial for advancing our understanding. Public health initiatives should also consider incorporating sleep-related interventions within tobacco control programs. Overall, such efforts in public health strategies will curb the escalating prevalence of e-cigarette use and safeguard the sleep health of the population.

It is also important to consider the possibility of reverse or bi-directional associations. Individuals experiencing poor sleep or high stress levels may be more likely to use e-cigarettes as a coping mechanism. In this context, sleep disturbances may precede or exacerbate e-cigarette use, rather than result from it. Future longitudinal studies are needed to study the directionality of these associations.

### Strengths and limitations

This study exhibits several strengths, including the incorporation of a comprehensive dataset from 16 cross-sectional studies involving 150730 exclusive e-cigarette user participants and a rigorous analytical approach assessing impact on sleep health. Most of the included studies statistically adjusted for key confounders such as age, sex, and socioeconomic status, which strengthens the reliability of the pooled estimates. However, the degree of adjustment varied across studies, particularly regarding health-related or behavioral factors, and this variability should be considered when interpreting the findings.

The age-stratified analysis further contributes to understanding the nuanced relationship between e-cigarette use and sleep health across different demographic groups. The sensitivity analysis showed that no single study disproportionately influenced the overall estimate, indicating robustness of our findings.

Nonetheless, several limitations exist, including the predominance of cross-sectional designs that limit causal inference and the geographical concentration of studies within the United States. Substantial heterogeneity observed across analyses also warrants cautious interpretation. We included one study reporting PR in the meta-analysis, whereas the remaining studies reported OR. However, sensitivity analysis showed that excluding the study reporting a PR did not materially change the overall pooled estimate (OR=1.38; 95% CI: 1.22–1.57). In addition, grey literature was excluded, which may introduce publication bias, although funnel plot and Egger’s test did not indicate significant bias. Our findings are based on self-reported measures and should therefore be interpreted with caution. Although some studies^15,33^ used validated instruments to assess sleep outcomes, such as the Pittsburgh Sleep Quality Index (PSQI), none of the included studies employed objective measures of sleep (e.g. polysomnography) or objective assessment of e-cigarette use (e.g. urine cotinine measurement). Consequently, most studies received negative ratings for Items 3 and 4 in Table 1. Future research should incorporate objective sleep assessments and, where possible, utilize longitudinal or experimental study designs to strengthen the evidence regarding the relationship between e-cigarette use and sleep health. Moreover, our assessment focused on non-smoker populations; future studies should compare exclusive e-cigarette users with dual users and conventional smokers to better clarify relative risks. Finally, since all the included studies were cross-sectional, the direction of the association between poor sleep and e-cigarette use cannot be established.

Overall, this study provides an important foundation for future research, underscoring the need for longitudinal designs, objective measurement of outcome, and further meta-analyses exploring the complex interrelationships between e-cigarette use, mental health, and sleep outcomes.

## CONCLUSIONS

Our systematic review and meta-analysis of 16 cross-sectional studies reveals a significant association between e-cigarette use and worsened sleep health, with an increased likelihood of poor sleep among past 30-day exclusive e-cigarette users, and this impact persists for all individuals irrespective of age. Despite study strengths, there are limitations such as predominance of cross-sectional designs that limit causal inference, and potential geographical concentration of studies within the United States. Future research should focus on longitudinal studies with objective sleep measures to better understand the intricate associations and role of potential mediators between e-cigarette use and sleep outcomes. As the landscape of nicotine delivery systems evolves, informed public health strategies and regulatory measures are needed to address the multifaceted implications of e-cigarette use on sleep health and overall well-being.

## Supplementary Material



## Data Availability

The data supporting this research are available from the authors on reasonable request.
