# Supplementary material for: The association between e-cigarette use and sleep health: A systematic review and meta-analysis
Source: Tob Induc Dis. 2026 Sep 18;24:10.18332/tid/225458. doi: 10.18332/tid/225458 (PMC13588213; doi:10.18332/tid/225458)
Supplement: Supplementary file 1 [file TID-24-168-s1.pdf]

Tobacco Induced Diseases

Supplementary file

© 2026 Gulati I. et al.

DOI:
10.18332/tid/225458

The content has been provided by the author(s) and has not been reviewed, verified, or endorsed by European Publishing. It may not have undergone peer review. The views, opinions, and recommendations expressed are solely those of the author(s) and do not necessarily reflect the position of European Publishing. European Publishing accepts no responsibility or liability for any consequences arising from the use of, or reliance on, this content.

Supporting information 1. Database search strategies

Database	Search Strings
Ovid MEDLINE(R)	1. exp Sleep, REM/ or exp Sleep-Wake Transition Disorders/ or exp Sleep Duration/ or sleep.mp. or exp Sleep Apnea, Central/ or exp Sleep Disorders, Intrinsic/ or exp Sleep Aids, Pharmaceutical/ or exp Sleep/ or exp Sleep Latency/ or exp REM Sleep Behavior Disorder/ or exp Sleep Stages/ or exp Sleep Disorders, Circadian Rhythm/ or exp Sleep Arousal Disorders/ or exp Sleep Phase Chronotherapy/ or exp Sleep Deprivation/ or exp Sleep Apnea, Obstructive/ or exp "Sleep Initiation and Maintenance Disorders"/ or exp Sleep, Slow-Wave/ or exp Sleep Wake Disorders/ or exp REM Sleep Parasomnias/ or exp Sleep Paralysis/ or exp Sleep Hygiene/ or exp Sleep Apnea Syndromes/ or exp Delta Sleep-Inducing Peptide/ or exp Sleep Quality/ or exp Sleep Medicine Specialty/ or exp Sleep Bruxism/<br>2. (e-cig* or electronic cigarette* or JUUL* or electronic vapour product* or alternative nicotine delivery system* or ecigarette* or e-hookah* or vape pen* or vape* or electronic nicotine delivery system*).tw,kf.<br>3. exp Electronic Nicotine Delivery Systems/<br>4. exp Vaping/<br>5. 2 or 3 or 4<br>6. 1 and 5<br>7. limit 6 to (english language and humans)
APA PsycInfo	1. exp Vaping/<br>2. exp Electronic Cigarettes/<br>3. exp Sleep, REM/ or exp Sleep-Wake Transition Disorders/ or exp Sleep Duration/ or sleep.mp. or exp Sleep Apnea, Central/ or exp Sleep Disorders, Intrinsic/ or exp Sleep Aids, Pharmaceutical/ or exp Sleep/ or exp Sleep Latency/ or exp REM Sleep Behavior Disorder/ or exp Sleep Stages/ or exp Sleep Disorders, Circadian Rhythm/ or exp Sleep Arousal Disorders/ or exp Sleep Phase Chronotherapy/ or exp Sleep Deprivation/ or exp Sleep Apnea, Obstructive/ or exp "Sleep Initiation and Maintenance Disorders"/ or exp Sleep, Slow-Wave/ or exp Sleep Wake Disorders/ or exp REM Sleep Parasomnias/ or exp Sleep Paralysis/ or exp Sleep Hygiene/ or exp Sleep Apnea Syndromes/ or exp Delta Sleep-Inducing Peptide/ or exp Sleep Quality/ or exp Sleep Medicine Specialty/ or exp Sleep Bruxism/<br>4. (e-cig* or electronic cigarette* or JUUL* or electronic vapour product* or alternative nicotine delivery system* or ecigarette* or e-hookah* or vape pen* or vape* or vaping* or electronic nicotine delivery system*).mp. [mp=title, abstract, heading word, table of contents, key concepts, original title, tests & measures, mesh word]<br>5. 1 or 2 or 4<br>6. 3 and 5<br>7. limit 6 to (human and english language)
Embase Classic + Embase	1. exp Sleep, REM/ or exp Sleep-Wake Transition Disorders/ or exp Sleep Duration/ or sleep.mp. or exp Sleep Apnea, Central/ or exp Sleep Disorders, Intrinsic/ or exp Sleep Aids, Pharmaceutical/ or exp Sleep/ or exp Sleep Latency/ or exp REM Sleep Behavior Disorder/ or exp Sleep Stages/ or exp Sleep Disorders, Circadian

	Rhythm/ or exp Sleep Arousal Disorders/ or exp Sleep Phase Chronotherapy/ or exp Sleep Deprivation/ or exp Sleep Apnea, Obstructive/ or exp "Sleep Initiation and Maintenance Disorders"/ or exp Sleep, Slow-Wave/ or exp Sleep Wake Disorders/ or exp REM Sleep Parasomnias/ or exp Sleep Paralysis/ or exp Sleep Hygiene/ or exp Sleep Apnea Syndromes/ or exp Delta Sleep-Inducing Peptide/ or exp Sleep Quality/ or exp Sleep Medicine Specialty/ or exp Sleep Bruxism/<br><br>2. exp Electronic Nicotine Delivery Systems/<br>3. exp Vaping/<br>4. (e-cig* or electronic cigarette* or JUUL* or electronic vapour product* or alternative nicotine delivery system* or ecigarette* or e-hookah* or vape pen* or vape* or vaping* or electronic nicotine delivery system*).tw,kf.<br>5. 2 or 3 or 4<br>6. 1 and 5 337<br>7. limit 6 to (human and English language)

Supporting information 2. Summary characteristics of the included studies (n=16)

Author (Year)	Aims	Country	Study design	Setting/ Methods	Study Years	Study Population (description, inclusion and exclusion)	Sample Size	Outcome	Exposure	Vaping Status	Statistical Methods	Key results	Significance
Wiener R.C et al. (2020)	The purpose of this study is to determine if there is an association between sleep duration and electronic cigarette use.	United States	Cross sectional study	Online survey	2015-2016	Non-institutionalized US residents, adults aged 18-65 years	2889	Sleep duration	Self-reported exposure: electronic cigarette use outcome: sleep duration	Any use over past 30 days	Descriptive, chi-square test, bivariate and multinomial logistic regression	Participants who currently use electronic cigarettes were 1.82 times more likely not to get their recommended sleep time duration as compared with participants who never used electronic cigarettes.	Significant
Merianos A.L. et al. (2023)	This study assessed the associations between current exclusive e-cigarette use, exclusive cigarette	United States	Cross sectional study	Telephone-based health survey	2020	Non-institutionalized US residents, young adults ages 18-24 years	13978	Sleep duration	Self-reported exposure: exclusive e-cigarette use vs exclusive cigarette use	Use every day or some days	Descriptive statistics, chi-square tests, weighted unadjusted logistic regression model	Exclusive e-cigarette users were at 41% increased odds of reporting inadequate sleep	Significant

	smoking, and dual e-cigarette and combustible cigarette use and inadequate sleep duration among US young adults												duration compared to non-users, with a p value less than 0.00, while adjusting for the covariates.<br>NS difference between exclusive e-cigarette and exclusive cigarette users.	
Dunbar M et al (2017)	Examining how the health profiles of youth that report ec use compare to those of youth who use cigarettes only, use both cigarettes and e-cigarettes (dual users), or use neither type of product.	United States	Cross sectional study	Web-based survey	2015	9th to 11th graders from 16 middle schools in the Greater Los Angeles area	2488	Sleep<br>Quality; sleep duration	Self-reported exposure: e-cigarette use	Use in the past year	Descriptive, ANOVA, bivariate logistic regression, ordinary least squares (OLS) regression models	E-cigarette-only users reported 0.27 hours ($p<0.01$) shorter weekend sleep times. No difference in sleep quality.	Not significant

Riehm K et al. (2019)	The objective of this study was to assess whether exclusive e-cigarette, exclusive combusted cigarette, and dual-product use are associated with sleep-related complaints among adolescents.	United States	Cross sectional study	Audio computer-assisted self-interviewing	2013 – 2015	Adolescents living in the U.S, aged 12-17, who had no missing data, if missing data was there present on at least one variable required for the analysis variable excluded	9588	Sleep complaints	Self-reported exposure: e-cigarette use outcome: sleep complaints	Use in the past year	Descriptive, adjusted and unadjusted logistic regression	The association between sleep-related complaints and e-cigarette were statistically significant and 60% higher among e-cigarette users compared to non-users	Significant
Brett E et al. (2020)	The current study aimed to determine the impact of e-cigarette use on self reported sleep health among young adults in college	United States	Cross sectional study	Online	Not available	Undergraduate students from a large midwestern university	1664	Sleep quality ; sleep latency, sleep duration	Exposure: e-cigarette use outcome: sleep quality	Use in the past 30-day e-cigarette users	Descriptive, ANOVA, multivariate analysis (MANOVA)	Global sleep scores were significantly lower, with a mean difference of 5.51, among never users compared to daily e-cigarette users.<br>No significant difference in sleep latency and duration between never user and daily e-	Significant

												cigarette users.	
Jackson D et al. (2020)	This paper addresses this gap in the literature by examining associations between vaping (nicotine or marijuana) and poor diet, poor sleep quality, and low levels of exercise	United States	Cross sectional study	Online survey	2017	Young adolescents (grades 8 and 10) enrolled in American schools	7765	Sleep duration	Exposure: nicotine vaping (no cigarette use)] outcome: sleep duration	Use in past 30 days	Descriptive, logistic and binomial regression	Youths who vaped had 5% higher odds of short sleep duration outcomes relative to abstaining youths.	Not significant
Kianersi S et al. (2021)	To determine whether e-cigarette use is associated with sleep deprivation in a sample of young (18 to 24 years-old) American adults.	United States	Cross sectional study	Random-digit dialing	2017 and 2018	Young adults aged 18 to 24 forty-one states and U.S territories. excluded states and territories that did not include the optional modules of e-cigarette/sleep deprivation in the questionnaire.	19701	Sleep duration	Exposure: e-cigarette use outcome: sleep quality/ duration	Everyday use	Descriptive, Poisson regression model, prevalence ratios (pr)	After adjusting for the confounders, compared to never e-cigarette users everyday e-cigarette smokers were 42% more likely to self-report sleep deprivation.	Significant
Merianos A.L et al. (2021)	The study aimed to investigate the relationships between	United States	Cross sectional study	School-based survey	2017	Students enrolled in grades 9-12 at public and private schools	11296	Sleep duration	Exposure: use of e-cigarettes outcome:	Use in past 30 days	Descriptive, chi-square tests, unadjusted and	Compared to no use, exclusive e-cigarette use is	Significant

	current exclusive e-cigarette use, exclusive combustible cigarette smoking, and dual use of e-cigarettes and combustible cigarettes, and insufficient sleep among US adolescents.					in the US, 13-18 years old. excluded individuals with missing information on or who used cigars and/or smokeless tobacco			sleep duration		adjusted logistic regression	consistently associated with 61% higher odds of insufficient sleep among US adolescents defined by the American academy of sleep medicine.<br>NS difference between exclusive e-cigarette vs exclusive cigarette users.	
Benyo S et al. (2021)	The objectives of our study were to examine the frequency of e-cigarette use; to identify demographic characteristics, risk-taking behaviours, and beliefs about vaping; and to determine symptoms associated	United States	Cross sectional study	Penn State Hershey Medical Center (Dauphin County, Pennsylvania)	2019-2020	Adolescents and young adults from emergency department and outpatient pediatric clinic, English-speaking, aged 12 to 23. excluded those who presented with altered mental status, severe pain, acute	494	Sleep disturbance	Exposure: the use of e-cigarette vs. non-users outcome: sleep disturbance	Use in the past month	Descriptive statistics, risk differences, Pearson-statistic, fisher-exact tests, and 2 sample t-tests	There is a 81% higher risk of a sleep disturbance occurring in an individual who uses e-cigarettes compare to those who are non-users	Significant

	with e-cigarettes among adolescents					psychiatric illness, respiratory distress, had a history of developmental delay, or were non english speaking.							
Advani I et al. (2022)	The study aims to assess the impact of e-cigarette, conventional tobacco, and dual use on sleep quality, sleep latency, cough, and drug use	Other: 47 countries across 6 continents	Cross sectional study	Online	2019-2020	Inhalant users, aged 13 and above, who completed the entirety of the survey with valid responses	1198	Sleep quality ; sleep latency	Exposure: use of e-cigarette vs. non-smokers outcome: sleep quality	Use in the past month	Descriptive, ANOVA, chi-square, multivariate linear regression, mean difference, logistic regression	Did not find any significant associations between overall sleep quality, as measured by PSQI scores, and inhalant usage<br><br>NS difference between exclusive e-cigarette vs exclusive cigarette users.	Not significant
Smucker, S et al (2023)	To investigate whether active component service members who use both e-cigarettes and other tobacco products are	United States	Cross sectional study	Survey	2018	Active component services members, as of September 2018. excluded service academies, senior military	17166	Sleep duration	Exposure: use of e-cigarettes outcome: sleep duration	Use in the past month	Descriptive, logistic regression	The odds of insufficient sleep are 1.09 times higher for those who only use e-	Not significant

	more likely to engage in other types of substance use or negative health behaviors.					colleges, and other reserve officers training corps programs and service members of flag rank (i.e., those above o-6). reserve members are excluded from the analysis as they are subject to military rules and regulations regarding the use of tobacco products only when on an activated status.						cigarettes to no use.<br>NS difference between exclusive e-cigarette and exclusive cigarette users.	
Baiden P et al. (2023)	To investigate the cross-sectional association between the use of e-cigarettes and insufficient sleep among adolescents.	United States	Cross sectional study	Public and private schools in the us	2017 and 2019	Youth in grades 9 to 12, aged 13 to 18	28 442	Sleep duration	Exposure: use of electronic vapor product outcome: sleep duration	Use in the past month	Descriptive, multicollinearity, binary logistic regression	Compared to no use, current use of e-cigarettes was associated with 33% increased odds of insufficient sleep	Significant

Christian W et al. (2023)	This study investigated the relation between e-cigarette and cigarette use and sleep duration in Kentucky, a state with high rates of nicotine dependence and related chronic diseases	United States	Cross sectional study	Telephone survey	2016 and 2017	Kentucky adults aged 18 years and older	18907	Sleep duration	Exposure: current use of e-cigarettes outcome: sleep duration	Use everyday or some days	weighted prevalence estimates and unweighted frequencies, prevalence odds ratio using weighted poisson regression ana lysis	Those who currently used e-cigarettes only were similarly likely (OR = 0.99) to sleep <7 hours per night compared with those who never smoked cigarettes or used e-cigarettes.	Not significant
Li et al. (2024)	To examine associations between e-cigarette use and inadequate sleep duration among adults and adolescents using the 2022 Canadian Community Health Survey data.	Canada	Cross-sectional	Population-based national survey	2022	Adults and youth aged 12 years and older	8964	Sleep duration (less than 7 hours per night)	Current e-cigarette use vs. never users	Use in past 30 days	Multivariable logistic regression adjusted for age, sex, education, and substance use	E-cigarette users had 1.42 times higher odds of insufficient sleep compared to never users ($p < 0.01$).	Significant
Poudel et al. (2024)	To assess the relationship between dual e-cigarette and cigarette use and poor sleep health among	United States	Cross-sectional	2020 National Health Interview Survey	2020	Adults aged 18–34 years	7320	Poor sleep quality	Past 30-day dual use of e-cigarettes and cigarettes	Past 30-day use	Weighted logistic regression adjusting for demographics	E-cigarette users and dual users had significantly higher odds of reporting	Significant

	young adults in the United States.										and mental health variables	poor sleep compared to non-users (OR = 1.32, 95% CI: 1.06-1.66; OR= 1.81, 95% CI: 1.46–2.24).	
Wang et al. (2023)	To evaluate the association between e-cigarette use and sleep problems among college students in China.	China	Cross-sectional	Web-based survey across five universities	2023	Undergraduate students aged 18–25 years	2415	Sleep disturbance (Pittsburgh Sleep Quality Index > 5)	Current e-cigarette use vs. non-users	Use in the past 30 days	Binary logistic regression	Current users were 1.55 times more likely to report poor sleep quality compared to non-users.	Significant

Abbreviations: ANOVA, Analysis of variance; MANOVA, Multivariate analysis of variance; OR, Odds ratio; PSQI, Pittsburgh Sleep Quality Index; US, The United States.

Supporting information 3. Meta-regression examining age group as moderator of the association between e-cigarette use and poor sleep health

**Moderator variable**	**Co-efficient (95% CI)**	**Q-test for moderators: QM (df)**	**P-value**
Age group (adolescents vs adults; reference: adolescents)	0.029 (-0.259 - 0.317)	0.04 (1)	0.844

Supporting information 4

Figure S1. Funnel plot assessing potential publication bias among studies (n=12) included in the meta-analysis

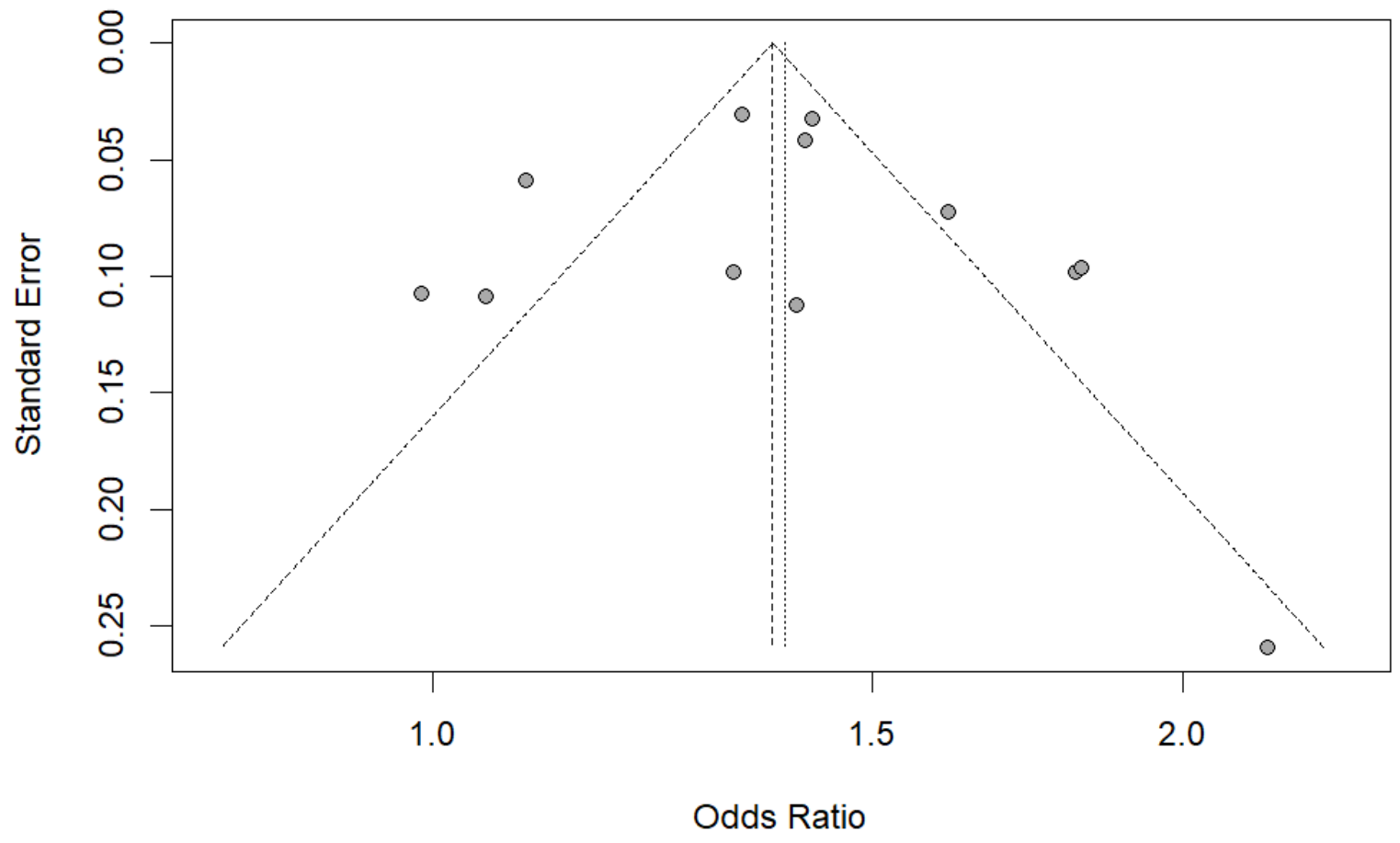

Figure S2. Leave-one-out sensitivity analysis evaluating the influence of individual studies (assessing association of exclusive e-cigarette use and poor sleep health) on the pooled Odds Ratio estimate (OR) with 95% Confidence Interval (CI). Estimates were pooled using random effects model

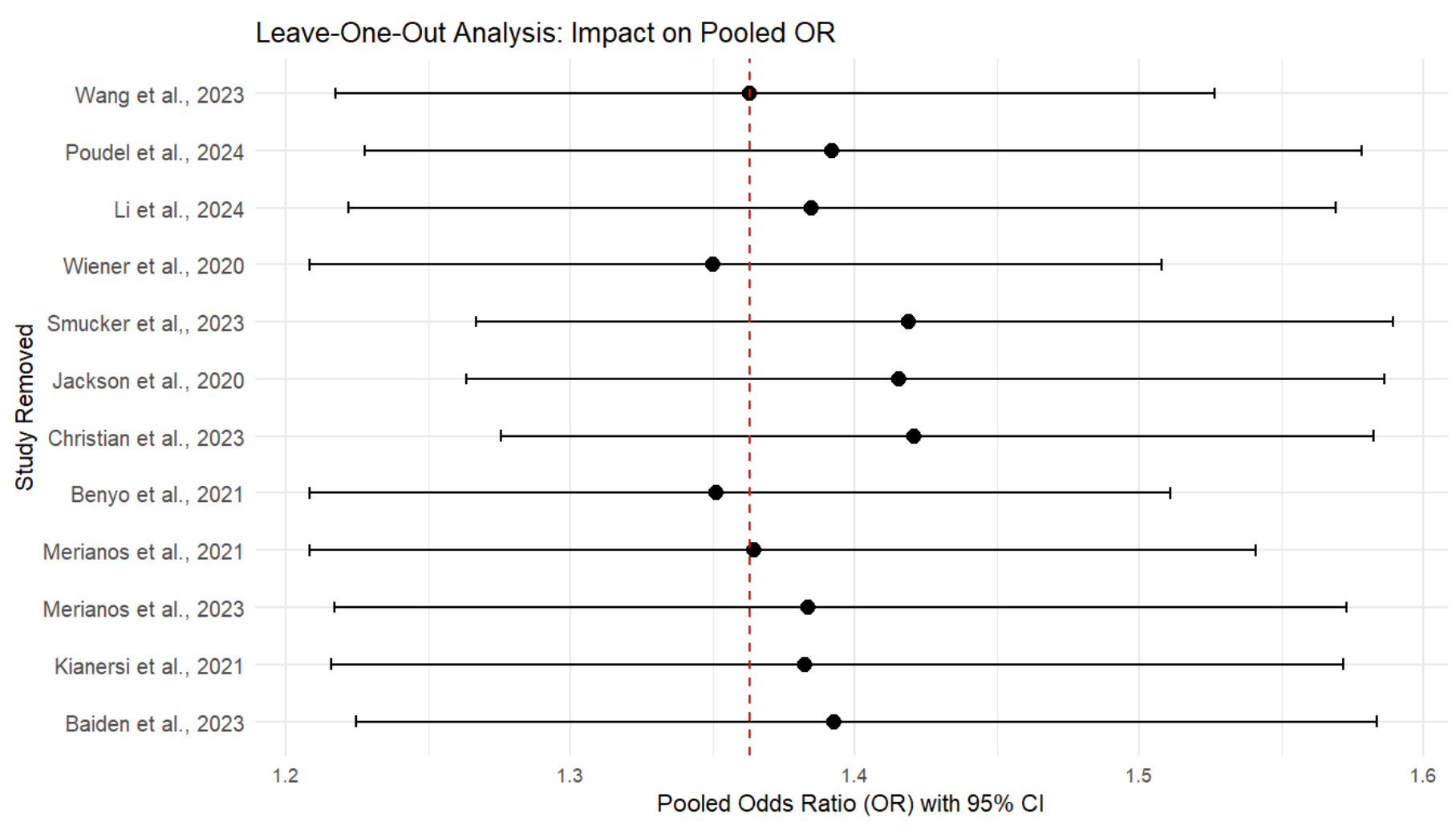

Supporting information 5. Sensitivity analysis findings (impact of excluding each study on the impact on poor sleep health)

**Excluded Study Author (Year)**	**Pooled Odds Ratio (OR)**	**95% Confidence Interval (CI)**	I^2	**P-Value (Q-test)**
Baiden et al., 2023	1.39	1.22-1.58	82%	<0.001
Kianersi et al., 2021	1.38	1.22-1.57	82%	<0.001
Merianos et al., 2023	1.38	1.22-1.57	83%	<0.001
Merianos et al., 2021	1.36	1.21-1.54	81%	<0.001
Benyo et al., 2021	1.35	1.21-1.51	80%	<0.001
Christian et al., 2023	1.42	1.28-1.58	79%	<0.001
Jackson et al., 2020	1.42	1.26-1.59	81%	<0.001
Smucker et al., 2023	1.42	1.27-1.59	76%	<0.001
Weiner et al., 2020	1.35	1.21-1.51	80%	<0.001
Li et al., 2024	1.38	1.22-1.57	83%	<0.001
Poudel et al., 2024	1.39	1.23-1.58	83%	<0.001
Wang et al., 2023	1.36	1.22-1.53	82%	<0.001
